# Repair on the Go: *E. coli* Maintains a High Proliferation Rate while Repairing a Chronic DNA Double-Strand Break

**DOI:** 10.1371/journal.pone.0110784

**Published:** 2014-10-29

**Authors:** Elise Darmon, John K. Eykelenboom, Manuel A. Lopez-Vernaza, Martin A. White, David R. F. Leach

**Affiliations:** Institute of Cell Biology, University of Edinburgh, Edinburgh, United Kingdom; National Cancer Institute, United States of America

## Abstract

DNA damage checkpoints exist to promote cell survival and the faithful inheritance of genetic information. It is thought that one function of such checkpoints is to ensure that cell division does not occur before DNA damage is repaired. However, in unicellular organisms, rapid cell multiplication confers a powerful selective advantage, leading to a dilemma. Is the activation of a DNA damage checkpoint compatible with rapid cell multiplication? By uncoupling the initiation of DNA replication from cell division, the *Escherichia coli* cell cycle offers a solution to this dilemma. Here, we show that a DNA double-strand break, which occurs once per replication cycle, induces the SOS response. This SOS induction is needed for cell survival due to a requirement for an elevated level of expression of the RecA protein. Cell division is delayed, leading to an increase in average cell length but with no detectable consequence on mutagenesis and little effect on growth rate and viability. The increase in cell length caused by chronic DNA double-strand break repair comprises three components: two types of increase in the unit cell size, one independent of SfiA and SlmA, the other dependent of the presence of SfiA and the absence of SlmA, and a filamentation component that is dependent on the presence of either SfiA or SlmA. These results imply that chronic checkpoint induction in *E. coli* is compatible with rapid cell multiplication. Therefore, under conditions of chronic low-level DNA damage, the SOS checkpoint operates seamlessly in a cell cycle where the initiation of DNA replication is uncoupled from cell division.

## Introduction

Unrepaired DNA double-strand breaks (DSBs) are a lethal form of damage. In *Escherichia coli*, DNA double-strand break repair (DSBR) is carried out by homologous recombination, a pathway that has been conserved in evolution from bacteria to humans. Recombination mediates repair of a damaged DNA molecule using an undamaged template, which is usually the sister chromosome generated during DNA replication [1]. This reaction is centrally catalyzed by the RecA protein in *E. coli* and by its homologue Rad51 in eukaryotes [2]. DNA damage is also used as a signal to alter a cellular pathway controlling cell division and DNA repair, known as a DNA damage checkpoint. Inhibition of cell division is believed to allow time for DNA repair to occur [3], [4]. In *E. coli*, the main DNA damage checkpoint is the SOS response [5]. It is controlled by the same RecA protein that mediates homologous recombination. In the presence of damaged DNA, RecA forms protein filaments on single-stranded DNA, which catalyze auto-cleavage and deactivation of the LexA protein that normally represses genes involved in DNA repair and cell survival [6], [7], [8]. Inhibition of cell division during the SOS response is mediated by the SfiA protein (also called SulA) [9], [10], [11]. SfiA inhibits the assembly of FtsZ, which is a tubulin-like protein essential at the early stage of cell division [12], [13], [14]. FtsZ polymerizes to form a ring-like structure at mid-cell where it acts as a scaffold for other division proteins. Another system that inhibits cell division is nucleoid occlusion, which is mediated by the SlmA protein that prevents the polymerization of FtsZ filaments into productive FtsZ rings in the presence of DNA [15], [16], [17].

The counterbalancing priorities of accurate DNA repair and rapid cell multiplication pose a potential dilemma to a unicellular organism. Does it have to sacrifice one in favor of the other? In eukaryotes, a delay in cell cycle progression is observed following induction of a high level of DNA damage [3], [4], [18], [19], [20]. Furthermore, in *Saccharomyces cerevisiae*, induction of checkpoint following chronic damage from low levels of UV light can lead to reduced cell viability [21]. In *E. coli*, induction of high levels of DNA damage can result in cell filamentation and death [22], [23], [24] but experiments using chronic low levels of DNA damage, which may be more frequently encountered under natural environmental conditions, are largely absent from the literature.

Previous work has shown that induction of an I-SceI endonuclease mediated DSB at a single locus in the *E. coli* chromosome can induce the SOS response [25], [26], [27]. However, that system has certain complexities. First, the I-SceI cleavage site is present on both sister chromosomes, so both chromosomes can be cleaved, which precludes repair. Second, at sites where repair is attempted from an intact sister chromosome, that has by chance escaped cleavage, the products of repair retain the cleavage site and can be re-cleaved. Third, if homologous DNA without the I-SceI recognition site (e.g. on an F′ plasmid) is provided to act as an intact non-sister DNA template, repair from this template will drive the loss of the I-SceI recognition site from the chromosome. These features of chromosome cleavage by I-SceI limit the applicability of this system for the study of chronic DNA breaks. Naturally, DSB repair by homologous recombination is often expected to occur following the formation of a DNA DSB on one chromosome in the presence of an intact sister chromosome. Therefore, the study of chronic DSBR at a single chromosomal location requires cleavage of only one sister chromosome and repair that does not eliminate the source of breakage. These conditions are satisfied by the system used in this study. A 246 bp interrupted palindrome has been introduced in the *E. coli* chromosome [28]. During each DNA replication cycle, this sequence can form a hairpin structure on the lagging-strand template. This structure is cleaved by the SbcCD hairpin endonuclease, leaving a two-ended DSB that is repaired by homologous recombination using the replicated leading strand as a template. In this experimental system, repair does not eliminate the palindrome [28], [29], [30], allowing a chronic breakage reaction to be established and studied in growing cells.

The present work investigates the consequences of a single chronic DNA DSB in the *E. coli* chromosome. Under these conditions, increased expression of RecA following SOS checkpoint induction is shown to be essential for cell survival. Strikingly, cells subjected to this DNA DSB are longer but their cell growth and viability are nearly unaffected. The roles of SfiA and SlmA proteins in the elongation of cells subjected to a chronic DSB were studied.

## Materials and Methods

### Strains and plasmids

**Figure 1:**
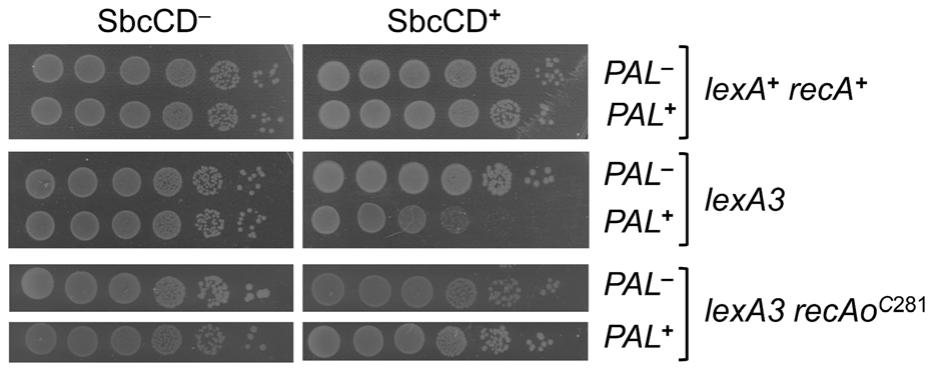
SOS-induced level of RecA is required for viability following DNA cleavage at a palindrome. Effect of SbcCD expression on the viability of *lexA3* and *lexA3 recAo^C^* ^281^ mutant strains encoding P*_BAD_-sbcDC* and containing or not the chromosomal 246 bp interrupted palindrome (*PAL*). Spot tests of ten-fold dilution series were carried out on LB plates complemented with either 0.2% of arabinose (SbcCD**^+^**) or 0.5% of glucose (SbcCD^-^).

A list of strains can be found in Table S1 in File S1. *E. coli* strains used were derivatives of BW27784 to allow a homogeneous expression from P*_BAD_*, when using the P*_BAD_-sbcDC* fusion (used for Figure 1), or otherwise were derivatives of MG1655 (please note that this MG1655 also has an *fnr-267* mutation) [31]. Mutations were introduced by P1 transduction or plasmid-mediated gene replacement [28]. Lists of plasmids and oligonucleotides can be found in Tables S2 and S3 in File S1, respectively, and the description of plasmid constructions are in Supplementary material.

### Growth of strains

For induction or repression of *sbcDC* expression, strains containing a P*_BAD_*-*sbcDC* fusion were grown in LB medium supplemented with 0.2% (w/v) arabinose or 0.5% (w/v) glucose, respectively. To measure the viability of *lexA3* and *lexA* **^+^** strains, 10-fold serial dilutions were prepared and 10 µl of these dilutions were spotted onto LB agar plates supplemented with 0.2% arabinose or 0.5% glucose. These experiments were carried out at least three independent times, giving similar results.

### Cell growth measurements

To measure growth levels, *sbcDC* **^+^** or Δ*sbcDC* cells containing or not the chromosomal 246 bp interrupted palindrome were grown at 37°C under agitation in LB medium. After an overnight culture, individual strains were diluted and maintained in exponential growth phase (OD_600nm_ <0.5; optical density readings at 600 nm) by appropriate dilution at regular intervals. Growth was monitored by measuring the OD_600nm_ and the number of cells was count by flow cytometry on samples taken every hour from these cultures. Samples were washed three times in sterile filtered PBS and numbers of cells per microliter were counted using an A50 Micro flow cytometer from Apogee Flow Systems. These experiments were carried out at least three independent times and the mean of generation times and numbers of cells were calculated (generation times were calculated using a doubling time program found on http://www.doubling-time.com).

### Competition experiment

After an overnight culture, individual strains were diluted to an OD_600nm_ of 0.01 in LB medium and grown for 2 hours at 37°C under agitation. Then, *PAL* **^+^** and *PAL* **^−^** strains were mixed in a ratio of 80% of *PAL* **^+^** for 20% of *PAL* **^−^**. In one set of experiments *PAL* **^+^** *yfp* **^+^** and *PAL* **^−^** *yfp* **^−^** strains were mixed together and in a second set *PAL* **^+^** *yfp* **^−^** and *PAL* **^−^** *yfp* **^+^** strains were mixed. These cell populations were diluted 10 times and grown in LB medium for 20 minutes and then diluted to an OD_600nm_ of 0.01. For the next 75 hours, cells were kept in exponential phase during the day (OD_600nm_ <0.5) by regular dilution to an OD_600nm_ of 0.01 in new LB medium and at night were either allowed to grow until stationary phase (ON^stat^) or kept at 4°C to stay in exponential phase (ON^4°C^). The OD_600nm_ of each culture was measured before and after every dilution and the number of cells per microliter expressing or not YFP was similarly monitored using flow cytometry after two washes in sterile filtered PBS. The cell fluorescence and number were measured using the blue excitation laser (488 nm) and detected on the green channel. PMT voltage parameters used were SALS  = 220, LALS  = 405, Blue  = 495, Green  = 500 and Red  = 520. Gain value parameters used were 1.0 for SALS, LALS, Blue and Green and 2.0 for Red. An example of flow cytometry results from one of these cultures is shown in Figure S1. The percentage of cells subjected to DSBs (*PAL* **^+^**) was calculated for each culture and time point. Please note that the *PAL* **^+^** cells will be the cell expressing the lowest fluorescence in *PAL* **^+^** *yfp* **^−^** and the cells expressing the higher fluorescence in the *PAL* **^+^** *yfp* **^+^** cells. The percentage of cells subjected to DSBs (*PAL* **^+^**) was calculated as 100 times the number of *PAL* **^+^** cells per microliter divided by the sum of *PAL* **^+^** and *PAL* **^−^** cells present per microliter of culture. For each time point, the percentage of cells subjected to DSBs (*PAL* **^+^**) was calculated before and after dilution of the culture and the mean between these two measurements was used to calculate the percentage of loss per generation of cells subjected to DSBs (*PAL* **^+^**). The number of generations between two time points was calculated on the assumption that N(t)  =  2^g(t)^ where N(t) is the number of cells at time t and g(t) is the number of generations that have elapsed at time t. The percentage of loss per generation of cells subjected to DSBs (*PAL* **^+^**) was the difference of percentage of cells subjected to DSBs (*PAL* **^+^**) between two time points divided by the number of generations between these two time points. In this study, we have assumed that the effect on growth of the presence of the 246 bp palindrome in the *lacZ* gene was independent from the effect on growth of the presence of the *yfp* gene into the *intC* gene. The results presented are the mean of three independent experiments.

### Rate of mutagenesis

The rate of mutation to rifampicin-resistance was measured by fluctuation analysis on 24 colonies for each *sbcDC* **^+^** or Δ*sbcDC* strains containing or not the chromosomal 246 bp interrupted palindrome [32]. After growth overnight of each colony at 37°C under agitation in liquid LB medium, appropriate dilutions of cells were plated onto LB agar plates or LB plates containing 100 µg/ml of rifampicin. Colony forming units were counted the next day. Bars presented in the graph show 95% confidence intervals. This experiment was carried out five independent times, giving similar results.

### SOS induction levels

SOS levels in cells containing the pGB150 plasmid (encoding a P*_sfiA_-gfp* fusion) were measured by microscopy. After an overnight culture, *sbcDC* **^+^** or Δ*sbcDC* cells containing or not the chromosomal 246 bp interrupted palindrome were diluted into fresh LB medium to an OD_600nm_ of 0.02 and grown for 80 minutes at 37°C under agitation (until an OD_600nm_ around 0.2). Cultures were then diluted ten times and grown again for 40 minutes until an OD_600nm_ around 0.1. Microscopy was performed to determinate the average Gray value of a line of pixels in 350 cells per strain (the mean of three lines of pixels taken at different places in the background was subtracted from each cell measurement in each picture). The data presented here are the mean of four independent experiments.

### Cell length measurements

Cell length in LB medium was measured by microscopy of early exponential phase *sbcDC* **^+^** or Δ*sbcDC* strains containing or not the chromosomal 246 bp interrupted palindrome. After an overnight culture, cells were diluted to an OD_600nm_ of 0.02 and grown for 80 minutes at 37°C under agitation (until an OD_600nm_ around 0.2). Cultures were then diluted ten times and grown again to an OD_600nm_ around 0.1. Data presented are the mean of four independent experiments investigating 350 cells each.

### Microscopy

10 µl of exponential phase cells were mounted on a bed of 1% agarose–H_2_O for viewing under the microscope. Images were acquired at a resolution of 0.1 µm per pixel using a Zeiss Axiovert 200 fluorescence microscope equipped with a Photometrics Evolve™ 512 EMCCD camera. Image acquisition and analyzes were carried out using the MetaMorph program (Molecular Devices). For each field of view, a single picture of the sharpest plane was taken and analyzed.

## Results

### SOS-induced level of RecA is required for the repair of a single DSB per replication cycle

The SOS response requirement for *E. coli* cell viability following the induction of a DSB by SbcCD at the site of a chromosomal 246 bp interrupted DNA palindrome was investigated. For this purpose, the *lexA3* mutation was introduced into strains expressing SbcCD under the control of an arabinose-inducible promoter (P*_BAD_*-*sbcDC*) in the presence or absence of the 246 bp interrupted palindrome at the chromosomal *lacZ* locus. The *lexA3* mutant gene encodes an uncleavable LexA protein that prevents the induction of the genes under the control of the SOS system [33]. As seen in Figure 1, the *lexA3* mutation conferred a viability decrease to cells subjected to a chronic DSB (SbcCD**^+^** *PAL* **^+^**). This result indicates that SOS induction is necessary for the survival of cells enduring a single DSB per replication cycle. Since the RecA protein, that is over-expressed during SOS induction [34], [35], is also essential for cell viability following SbcCD cleavage of the palindrome [28], we investigated whether cells subjected to this chronic DSB might need an elevated level of this protein. To determine whether an induced level of RecA protein was required for cell survival, the *recAo^C^* ^281^ mutation was introduced into the *lexA3* mutants [36]. In a *recAo^C^* ^281^ mutant, the LexA protein cannot bind the *recA* promoter, allowing a constitutively high level of RecA expression even in the absence of SOS induction. Notably, the *recAo^C^* ^281^ mutation completely rescued the low-viability phenotype of the *lexA3* mutant strain carrying the palindrome and expressing SbcCD (Figure 1). This finding demonstrates that an increased level of RecA expression is the only SOS-induced characteristic needed for viability of *E. coli* cells subjected to a chronic DSB using this system.

### A single repaired DSB per replication cycle induces the SOS response

**Figure 2:**
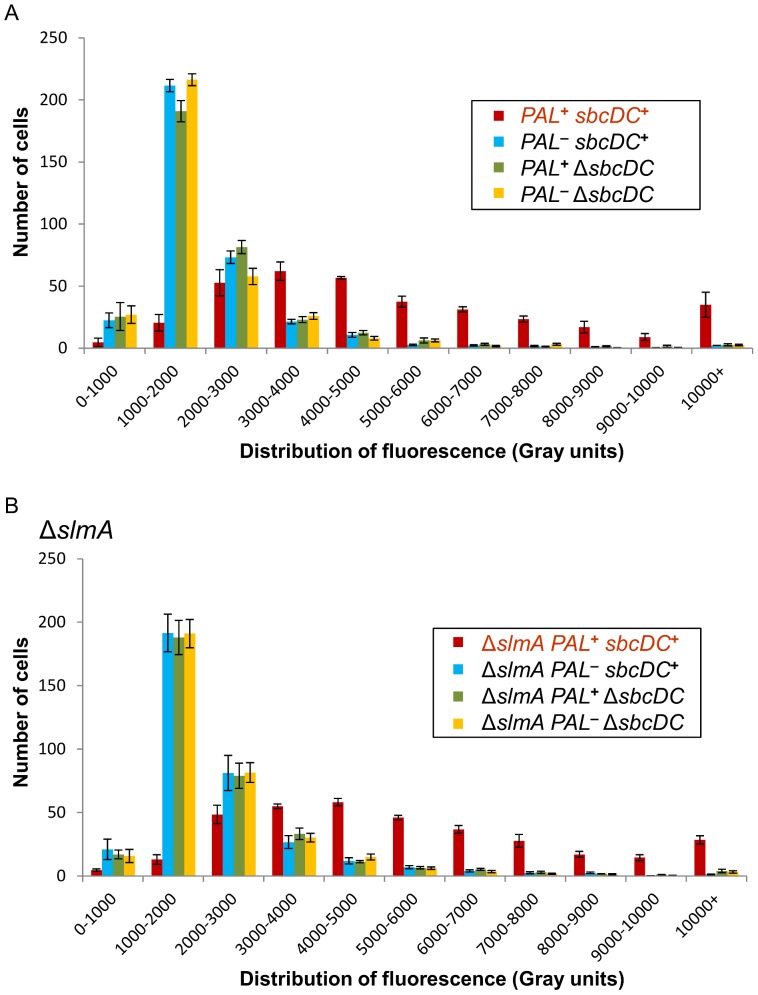
Cleavage of the 246 bp palindrome by SbcCD induces the SOS response. Fluorescence distribution profiles of average Gray value in pixels of populations of *sbcDC* **^+^** or Δ*sbcDC* cells containing or not the chromosomal 246 bp interrupted palindrome (*PAL*) and carrying a plasmid encoding a P*_sfiA_*-*gfp* fusion (pGB150). Error bars show the standard error of the mean of 4 independent experiments investigating 350 cells each. (A) In a wild-type background. (B) In a Δ*slmA* background.

The observation that an SOS induced level of RecA protein was required for cell survival implied the induction of the SOS response in populations of cells undergoing this chronic break. To measure the induction of the SOS system in individual cells, the fluorescence level was investigated by microscopy in *sbcDC* **^+^** and Δ*sbcDC* cells containing or not the 246 bp interrupted palindrome and carrying a plasmid containing the *gfp* gene under the control of the SOS-inducible *sfiA* promoter (P*_sfiA_*-*gfp*) (Figure 2A). The fluorescence level profiles were similar for palindrome-free *sbcDC* **^+^** and Δ*sbcDC* cells and for Δ*sbcDC* cells carrying the 246 bp palindrome. However, the fluorescence level increased dramatically in *sbcDC* **^+^** cells containing the 246 bp palindrome. Therefore, induction of a single targeted DSB per replication cycle significantly activates the SOS response.

### Rapid growth, good viability and low mutagenesis are maintained in populations of cells undergoing chronic DSBR once per replication cycle

**Figure 3:**
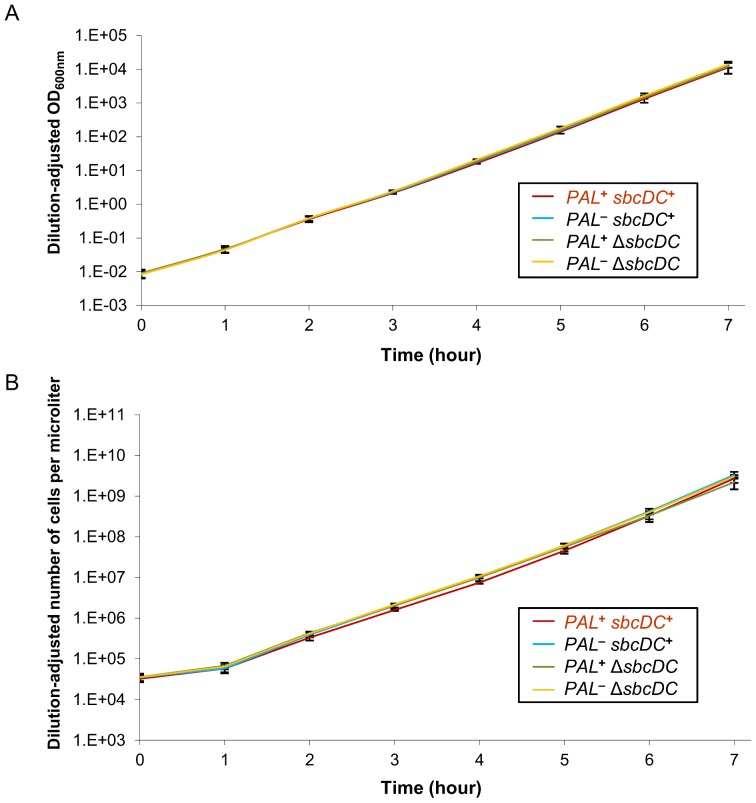
Cleavage of a palindrome by SbcCD has no detectable impact on cell growth. Graphs showing the growth level of *sbcDC* **^+^** and Δ*sbcDC E. coli* strains containing or not the chromosomal 246 bp interrupted palindrome (*PAL*). Error bars show the standard error of the mean of 3 independent experiments. (A) Dilution-adjusted optical density at 600 nm of cultures kept in exponential phase. (B) Dilution-adjusted average number of cells per microliter of cultures kept in exponential phase as counted by flow cytometry.

**Figure 4:**
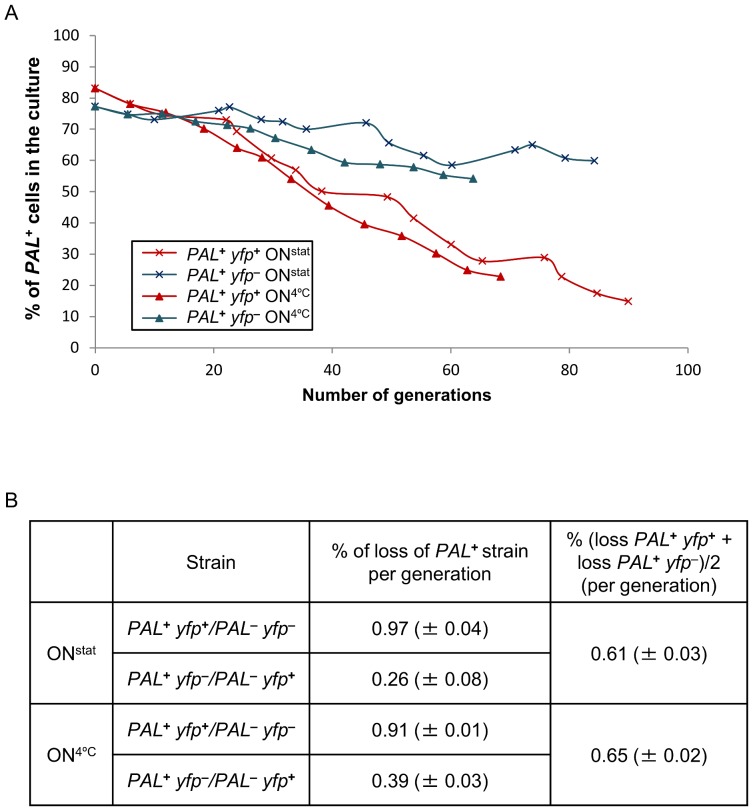
Effect of the cleavage of a palindrome by SbcCD quantified by competition experiments. Competition experiments were carried out between *PAL* **^+^** *yfp* **^+^** and *PAL* **^−^** *yfp* **^−^** strains on one hand and *PAL* **^+^** *yfp* **^−^** and *PAL* **^−^** *yfp* **^+^** strains on the other hand. Cells were either allowed to reach stationary phase at night time (ON^stat^; grown for more than 80 generations) or constantly kept in exponential phase (ON^4°C^; grown for more than 60 generations). (A) Example of a graph showing the percentage of cells containing the chromosomal 246 bp interrupted palindrome (*PAL*) in function of the number of generations of co-culture with a strain that does not contain the palindrome. These results are from the second replicate of the competition experiments. (B) Table presenting the average percentage of loss per generation of strains containing the chromosomal 246 bp interrupted palindrome (*PAL*) during these competition experiments. Errors indicated between brackets are the standard error of the mean of 3 independent experiments.

To investigate whether this level of chronic DSBR and induction of the SOS checkpoint lead to reduced growth and cell viability, cultures of *E. coli* cells were studied in the presence and absence of a chronic DSB. As shown in Figure 3 and Table 1, no differences were observed between growth rates or viabilities of *sbcDC* **^+^** and Δ*sbcDC* strains containing or not the palindrome over a period of 7 hours (corresponding to 24 generations). This result was confirmed by the observation that there was no detectable difference in colony size between any of the studied strains (Figure S2A). These experiments indicated that chronic DSBR must have little impact on cell growth or viability. However, a small growth disadvantage of cells undergoing DSBR (less than approximately 2% per generation) would not be detected in these experiments. Therefore, competition experiments were performed in which *sbcDC* **^+^** *PAL* **^+^** cells were mixed with *sbcDC* **^+^** *PAL* **^−^** cells and the relative proportions of the two cell types were evaluated over a longer period of cell growth (75 hours). The two populations of cells were differentially marked with a *yfp* gene (in both combinations of marking, to account for any effect of the *yfp* gene on cell viability) and cell numbers were counted by flow cytometry. As can be seen in Figure 4, cells undergoing chronic DSBR had a growth disadvantage of 0.6% per generation.

**Table 1:** *E. coli* generation time (minutes).

Background	*PAL* ^+^ *sbcDC* ^+^	*PAL* ^−^ *sbcDC* ^+^	*PAL* ^+^ Δ*sbcDC*	*PAL* ^−^ Δ*sbcDC*
Wild-type	19.7 (±1.2)	19.6 (±0.8)	19.5 (±0.3)	19.4 (±0.4)
Δ*sfiA*	21.2 (±0.9)	20.7 (±1.1)	21 (±0.4)	21.4 (±0.7)
Δ*slmA*	18.9 (±0.3)	16.5 (±1)	17.4 (±0.5)	17.5 (±0.9)
Δ*sfiA* Δ*slmA*	18 (±0.5)	17.2 (±0.9)	18.4 (±0.46)	18 (±0.4)

Errors indicated between brackets are the standard error of the mean of 3 independent experiments. No statistically significant differences (p-value <0.05) have been found between strains from the same background using an ANOVA two-factor with replication test (DSBs compared to no DSBs).

**Figure 5:**
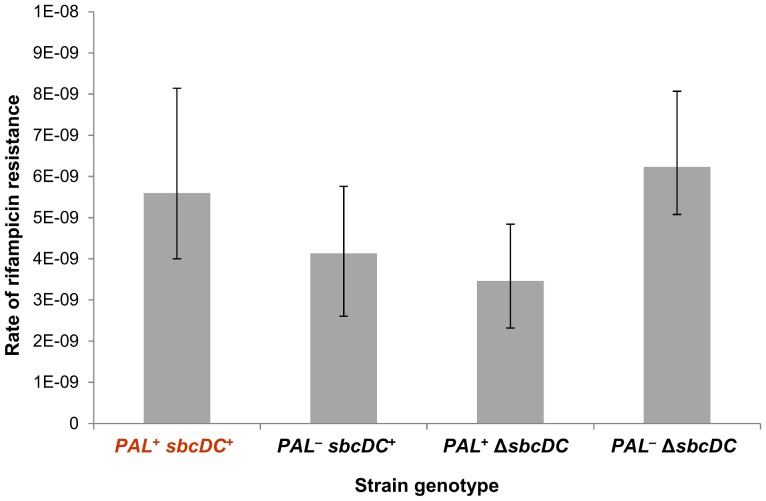
SOS induction following the cleavage of a palindrome by SbcCD does not induce mutagenesis. Fluctuation analysis measuring the rate of mutagenesis (mutation to rifampicin resistance cells) in *sbcDC* **^+^** and Δ*sbcDC E. coli* strains containing or not the chromosomal 246 bp interrupted palindrome (*PAL*). Error bars show the 95% confidence intervals.

Induction of the SOS response has the potential to increase mutagenesis due to the activation of one or both of the error-prone polymerases (PolIV and PolV) [37], [38], [39], [40], [41]. *rpoB* was used as a target gene to determine whether the induction of the SOS system in response to a single DSB per replication cycle leads to an increase in the level of mutagenesis. *rpoB* mutants were selected by their ability to grow in the presence of rifampicin and their rate of formation was determined by fluctuation analysis (Figure 5) [32]. No increase in mutation rate was observed in the strain undergoing chronic DSBR, indicating that chronic induction of the SOS response in strains containing the 246 bp palindrome and expressing SbcCD does not induce PolIV- or PolV-mediated mutagenesis.

### A single repaired DSB per replication cycle causes inhibition of cell division

**Figure 6:**
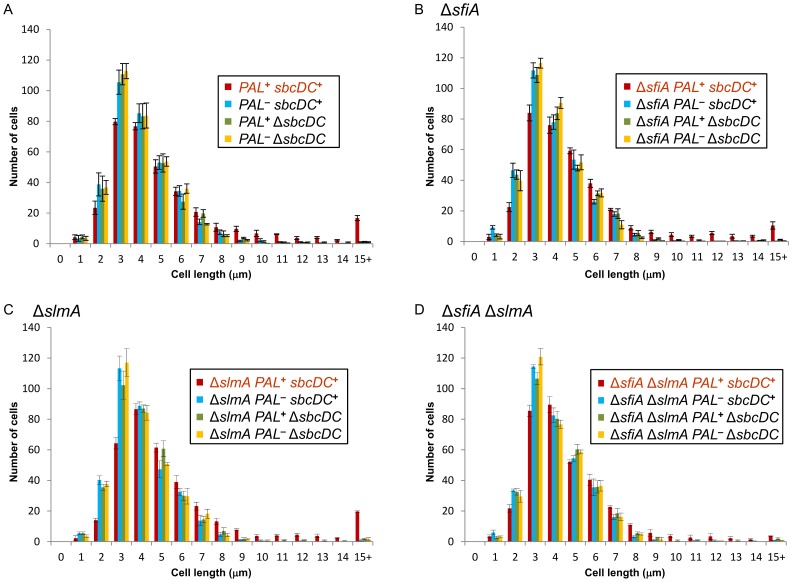
Role of SfiA and SlmA in the delay of cell division following a chronic DSB. Graphs displaying the cell length distribution in micrometers of *sbcDC* **^+^** and Δ*sbcDC E. coli* cells containing or not the chromosomal 246 bp interrupted palindrome (*PAL*). Error bars show the standard error of the mean of 4 independent experiments investigating 350 cells each. (A) In a wild-type background. (B) In a Δ*sfiA* background. (C) In a Δ*slmA* background. (D) In a Δ*sfiA* Δ*slmA* background.

To determine whether a single DSB per replication cycle inhibits cell division, the length of *sbcDC* **^+^** and Δ*sbcDC* cells containing or not the palindrome was measured. The mean length of *sbcDC* **^+^** cells containing the palindrome was almost 34% longer than that of cells lacking SbcCD and/or the palindrome (Table 2). The distribution of cell lengths was significantly different in the strain subjected to a chronic DSB (*PAL* **^+^** *sbcDC* **^+^**; Figure 6A; Tables 3, 4 and 5). The change in length distribution caused by chronic DSBR was characterized by two principal features, a decrease in the number of small cells (<4 µm in length; Table 3) and an increase in filamentation (defined here as cells longer than 8 µm; Table 4). About 17% of *sbcDC* **^+^** cells containing the palindrome were longer than 8 µm whereas only 4% of cells that were not subject to a chronic DSB reached that length. In addition, time-lapse microscopy was used to observe live filamentation of *sbcDC* **^+^** cells containing the palindrome (Videos S1, S2, S3 and S4 in Supplementary data).

**Table 2:** Average *E. coli* cell length (micrometers).

Background	*PAL* ^+^ *sbcDC* ^+^	*PAL* ^−^ *sbcDC* ^+^	*PAL* ^+^ Δ*sbcDC*	*PAL* ^−^ Δ*sbcDC*	% DSB^+^/DSB^−^
Wild-type	6.2 (±0.1) *	4.7 (±0.2)	4.7 (±0.1)	4.6 (±0.1)	133.8%
Δ*sfiA*	5.8 (±0.2) *	4.4 (±0.1)	4.6 (±0.1)	4.4 (±0.1)	129.7%
Δ*slmA*	6.5 (±0.1) *#	4.5 (±0.1)	4.7 (±0.1)	4.6 (±0.1)	140.6%
Δ*sfiA* Δ*slmA*	5.4 (±0.1) *#†	4.6 (±0.1)	4.8 (±0.1)	4.6 (±0.1)	115.1%

Errors indicated between brackets are the standard error of the mean of 4 independent experiments investigating 350 cells each.

* Statistically significantly different from the other strains in the same background using an ANOVA two-factor with replication test (DSBs compared to no DSBs; p-value <0.05).

# Statistically significantly different from the wild-type version of this strain using an unpaired t-test (p-value <0.05).

† Statistically significantly different from the Δ*slmA sfiA* **^+^** version of this strain using an unpaired t-test (p-value <0.05).

**Table 3:** Percentage of *E. coli* cells shorter than 4 µm (%).

Background	*PAL* ^+^ *sbcDC* ^+^	*PAL* ^−^ *sbcDC* ^+^	*PAL* ^+^ Δ*sbcDC*	*PAL* ^−^ Δ*sbcDC*	% DSB^+^/DSB^−^
Wild-type	30.7 (±1.2)	42.2 (±4.3)	43.2 (±4.1)	43.7 (±2.5)	71.3
Δ*sfiA*	31.3 (±2.7)	47.9 (±1.3)	44.8 (±2.2)	45.6 (±2)	67.9
Δ*slmA*	23 (±1.5)	45.4 (±2.8)	40.9 (±2.7)	45.2 (±2.2)	52.5
Δ*sfiA* Δ*slmA*	31.6 (±1)	44 (±0.5)	40.5 (±1)	43.9 (±2.4)	73.8

Errors indicated between brackets are the standard error of the mean of 4 independent experiments.

**Table 4:** Percentage of *E. coli* cells longer than 8 µm (%).

Background	*PAL* ^+^ *sbcDC* ^+^	*PAL* ^−^ *sbcDC* ^+^	*PAL* ^+^ Δ*sbcDC*	*PAL* ^−^ Δ*sbcDC*	% DSB^+^/DSB^−^
Wild-type	17.2 (±1.2)	4.4 (±0.9)	4.4 (±0.6)	3.1 (±0.5)	433.2
Δ*sfiA*	13.2 (±1.2)	2 (±0.4)	3.4 (±0.7)	1.6 (±0.2)	566.5
Δ*slmA*	16.9 (±1)	2.5 (±0.6)	4.1 (±0.8)	2.5 (±0.7)	557.7
Δ*sfiA* Δ*slmA*	9.8 (±1.6)	2.1 (±0.5)	3.9 (±0.3)	2.4 (±0.5)	350

Errors indicated between brackets are the standard error of the mean of 4 independent experiments.

**Table 5:** Percentage of *E. coli* cells longer than 15 µm (%).

Background	*PAL* ^+^ *sbcDC* ^+^	*PAL* ^−^ *sbcDC* ^+^	*PAL* ^+^ Δ*sbcDC*	*PAL* ^−^ Δ*sbcDC*	% DSB^+^/DSB^−^
Wild-type	4.8 (±0.5)	0.3 (±0.1)	0.4 (±0.1)	0.3 (±0.1)	1454
Δ*sfiA*	3 (±0.7)	0.1 (±0.1)	0.4 (±0.1)	0.1 (±0.1)	1500
Δ*slmA*	5.6 (±0.2)	0.3 (±0.2)	0.5 (±0.1)	0.5 (±0.2)	1302
Δ*sfiA* Δ*slmA*	1.1 (±0.1)	0.3 (±0.2)	0.6 (±0.3)	0.3 (±0.2)	275

Errors indicated between brackets are the standard error of the mean of 4 independent experiments.

Inhibition of cell division can be controlled by the SOS-induced cell division inhibitor SfiA [9], [11] or by nucleoid occlusion in which SlmA plays an important role [17], [42]. The potential role of SfiA in the elongation of cells subjected to a chronic DSB was investigated. Cell growth and cell length of *sbcDC* **^+^** and Δ*sbcDC* strains containing or not the palindrome were similar in presence or absence of SfiA (Tables 1 and 2; Figures 6B and S2B), suggesting that other pathways contribute to cell elongation in cells subjected to a chronic DSB. The potential role of SlmA in the elongation of cells subjected to a chronic DSB was also investigated. There was no significant difference in the growth of Δ*slmA sbcDC* **^+^** and Δ*slmA* Δ*sbcDC* strains containing or not the palindrome (Table 1 and Figure S2C). Surprisingly, the distribution of length of Δ*slmA* cells subjected to a chronic DSB showed that the whole population of cells was significantly longer in the absence of SlmA (Tables 2, 3, 4, 5 and Figure 6C), indicating that there is an increase in the unit size of these cells. This result suggests that the absence of SlmA induced a nucleoid-occlusion independent pathway that inhibits cell division. Importantly, the levels of the SOS response in Δ*slmA* cells subjected or not to a chronic DSB were similar to those in a wild-type background (Figure 2), demonstrating that this increase in cell size does not originate from a more elevated SOS response in cells subjected to a chronic DSB in the absence of SlmA. To determine whether the action of SfiA and SlmA were both responsible for cell elongation caused by a chronic DSB, cell growth and cell length were studied in Δ*sfiA* Δ*slmA* double mutants. The growth rate of Δ*sfiA* Δ*slmA* strains was unaffected when subjected to a chronic DSB (Table 1 and Figure S2D). However, the cell length of two subpopulations was significantly affected by the deletion of the *sfiA* gene in a Δ*slmA* strain subjected to a chronic DSB (Tables 2, 3, 4, 5 and Figure 6D). Firstly, the number of cells under 4 µm increased back to a similar number as in the *slmA* **^+^** strains subjected to a chronic DSB (around 31% of the cells in *slmA* **^+^** and Δ*sfiA* Δ*slmA* backgrounds compared to 23% in the Δ*slmA* background; Figure 6D and Table 3). Secondly, the number of very long cells (more than 15 µm) dropped in the Δ*sfiA* Δ*slmA* strain subjected to a chronic DSB (around 1.1% of the cells compared to 3–5.6% in the other backgrounds subjected to a chronic DSB and 0.1–0.6% in cells not subjected to a chronic DSB; Figure 6D and Table 5). These results indicate that the two pathways performed by SfiA and SlmA are together responsible for the majority of the very long cells (cells over 15 µm in length) observed in presence of a chronic DSB. In addition, the increase in cell size observed in the whole population in a Δ*slmA* strain subjected to a chronic DSB requires the presence of SfiA but is not due to an increase of the SOS response.

It is clear that DSB induction causes three separable effects on cell size. First, there is a reduction of the number of small cells that occurs irrespectively of inactivation of SfiA and SlmA. Second, there is a further reduction of the number of small cells in presence of SfiA but absence of SlmA. Third, there is an increase in very long cells that occurs in the presence of either SfiA or SlmA and is only significantly reduced in the double mutant.

## Discussion

This study demonstrates that a single, efficiently repaired, DSB per chromosome per replication cycle is sufficient to induce the SOS response of *E. coli* and that this induction is required for cell viability. The only requirement for the SOS response in the survival of cells following this level of chronic DNA damage is the induced expression of RecA protein. It is possible that the need for an elevated level of RecA protein reflects the repetitive nature of the damage induced since a naïve cell encountering a DSB will not have an induced level of RecA protein and normally survives the damage. The fact that the population of *E. coli* cells subjected to this level of DSB induces the SOS response is consistent with the observation that the level of spontaneous DSBR in cells that are not inducing the SOS response at the population level is lower than one break per replication cycle. Previous estimates are that spontaneous DSBR occurs at a frequency of less than 1% per generation, as measured by SOS induction [25], and that replication restart requiring DnaC (which includes DSBR events) occurs in 18% of replication cycles [43].

We show that chronic DSBR results in an increase in cell size consistent with delayed cell division. It has been proposed that, following DNA damage, inhibition of cell division allows time for successful repair to occur before cell division can proceed [9]. Our data on cell size argue that there are three separable effects of chronic DSB that are differentially affected by the SOS-induced cell division inhibition system mediated by SfiA and nucleoid occlusion mediated by SlmA. First, there is a decrease in the number of small cells (<4 µm in length) that is indicative of a larger size of the unit cell and is independent of SfiA and SlmA. Second, in the absence of SlmA and presence of SfiA, this decrease in the number of small cells is accentuated.Third, there is a DSB-induced large increase in length that is only significantly reduced in the absence of both cell division inhibition systems mediated by SfiA and SlmA. Recently, the existence of an SOS- and SlmA-independent pathway blocking cell division was revealed by Cambridge and collaborators [44]. Whether or not this is the same system as that causing an increase in the unit cell size observed here remains to be determined.

Importantly, despite the requirement for SOS induction in cell survival and the activation of the checkpoint by this level of DNA damage resulting in a delay in cell division, cells maintain 99.4% growth rate and viability accompanied by no increase in mutagenesis. The prokaryotic cell cycle has partially unlinked its DNA replication and cell division cycles by uncoupling the initiation of DNA replication from cell division while retaining the link between termination of DNA replication and cell division [45], [46], [47]. In this way, rounds of DNA replication can overlap in situations where the DNA replication cycle takes longer than the cell division cycle. The observation of a cell loss of 0.6% per generation subjected to a chronic DSBR has four implications. First, the period between rounds of initiation of DNA replication in cells undergoing chronic DSBR must be at least 99.4% of that in control cells. A greater difference in this period would result in a corresponding difference in the number of genomes produced that would over the generations affect the number of viable cells. Second, cells increase in mass at approximately the same rate irrespective of chronic DSBR. Therefore, there is no change in metabolism that is sufficient to substantially alter the accumulation of cell mass. Third, at least 99.4% of chromosomes are eventually distributed appropriately to daughter cells even if this may be delayed in some cells that are experiencing a delay in cell division. And fourth, the vast majority of cells in which cell division has been inhibited are fully viable. That all these implications are satisfied in cells that have chronically induced an essential DNA damage checkpoint reveals the seamless operation of the SOS system when cells are experiencing a low level of chronic damage. The present study confirms that *E. coli* has evolved a cell cycle where it can reconcile the imperative for rapid cell multiplication with the operation of a checkpoint designed to ensure repair of DNA damage prior to cell division. The partially unlinked nature of the DNA replication and cell division cycles implies that *E. coli* can delay cell division in response to chronic DSBR without substantially affecting the time interval between initiations of DNA replication and can manage the consequences of segregating its chromosomes whatever extra time may be required to undertake DNA repair.

In a wider context, it is interesting to compare the checkpoint strategies adopted under similar situations by eukaryotic cells, in which the DNA replication cycle is more closely tied to the cell division cycle. There, replication generally takes up a less significant period within the cell cycle and accommodation to chronic checkpoint induction may be possible via the alternative strategy of altering the lengths of G1, S and G2 phases of the cell cycle, while dividing at a higher cell mass. It is also possible for some DNA damage to be carried over from one cell cycle to another [48], [49], [50]. However, it has been shown that activation of the DNA damage checkpoint can be detrimental to *S. cerevisiae* survival in the presence of continuous low levels of DNA damage by UV irradiation [21]. By contrast, in the same organism, checkpoint function is required for optimal growth and colony formation following chronic checkpoint induction caused by humanized telomeres [51]. Overexpression of Rad24 induces checkpoint activation in *S. pombe*, increases cell size and reduces growth rate [52], [53] but the reduction in growth rate may not simply be due to checkpoint activation. To our knowledge, the possibility that eukaryotic cells might be able to delay cell division by chronic checkpoint activation and yet retain growth and viability associated with normal growth conditions remains open. Clearly, this would not be desirable in many cells of multicellular eukaryotes where rapid multiplication would be of no selective advantage and might be associated with pathogenic consequences (e.g. cancer).

## Supporting Information

Figure S1 **Example of flow cytometry results from a competition experiment.** Results from flow cytometry analyses of the third replicate of the competition experiment between a *PAL* **^+^** *yfp* **^−^** strain and a *PAL* **^−^** *yfp* **^+^** strain after 23.5 hours of growth (these cells were allowed to reach stationary phase at night time). (A) Visualisation and selection of cells in function of light scatter angles. The flow cytometer counted and displayed 500,000 particles. A heat map indicated the population density of these particles. Cells were selected (here 466,255 cells were encircled in region of interest 1). (B) Visualisation and selection of cells in function of their green fluorescence. *PAL* **^+^** *yfp* **^−^** cells were encircled in region of interest 2 whereas *PAL* **^−^** *yfp* **^+^** cells were encircled in region of interest 7. (C) Number of cells in function of fluorescence when gated by region of interest 1. The cells selected in region of interest 1 in panel A were separated here in function of their fluorescence so that it was possible to evaluate the number of *PAL* **^+^** *yfp* **^−^** cells indicated in region of interest 5 and *PAL* **^−^** *yfp* **^+^** cells indicated in region of interest 6. (D) Number of *PAL* **^+^** *yfp* **^−^** cells in the population. The cells selected in region of interest 2 in panel B were separated in function of their fluorescence so that it was possible to calculate the number of *PAL* **^+^** *yfp* **^−^** cells indicated in region of interest 4. (E) Number of *PAL* **^−^** *yfp* **^+^** cells in the population. The cells selected in region of interest 7 in panel B were separated in function of their fluorescence so that it was possible to calculate the number of *PAL* **^−^** *yfp* **^+^** cells indicated in region of interest 3. Characteristics of regions of interest are indicated under each panel; the numbers of cells per microliter of culture (Evt/μl) were used for subsequent data analyses.
(TIF)

Figure S2 ***E. coli*** **viability is not significantly affected by a chronic DSB.** Viability of *sbcDC* **^+^** and Δ*sbcDC E. coli* strains containing or not the chromosomal 246 bp interrupted palindrome (*PAL*). Spot tests of ten-fold dilution series were carried out on LB plates. (A) Wild-type background strain. (B) Δ*sfiA* background strain. (C) Δ*slmA* background strain. (D) Δ*sfiA* Δ*slmA* background strain.
(TIF)

File S1 **Main supporting information file.** This file includes additional materials and methods (strains and plasmids, spot test, time-lapse microscopy), additional results (time-lapse microscopy), Table S1 (*E. coli* strains), Table S2 (plasmids), Table S3 (oligonucleotides) and additional references.
(DOC)

Video S1 **PAL+ SbcCD+.**
(MOV)

Video S2 **PAL− SbcCD+.**
(MOV)

Video S3 **PAL+ SbcCD−.**
(MOV)

Video S4 **PAL− SbcCD−.**
(MOV)
