# Supplementary figures and images for: Repair on the Go: E. coli Maintains a High Proliferation Rate while Repairing a Chronic DNA Double-Strand Break
Source: PLoS One. 2014 Oct 29;9(10):e110784. doi: 10.1371/journal.pone.0110784 (PMC4213011; doi:10.1371/journal.pone.0110784)

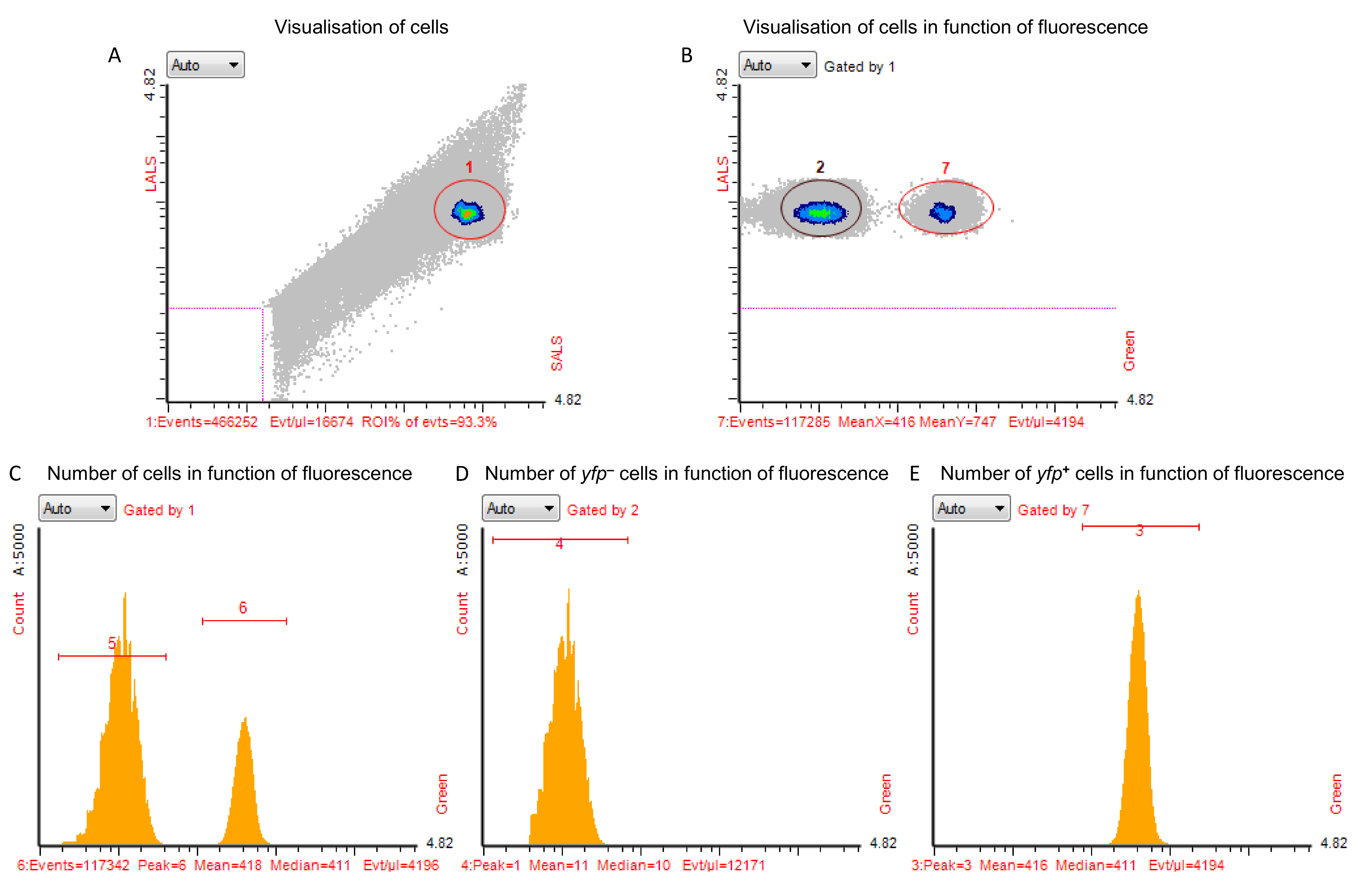

Supplement: Figure S1 — Example of flow cytometry results from a competition experiment. Results from flow cytometry analyses of the third replicate of the competition experiment between a PAL + yfp − strain and a PAL − yfp + strain after 23.5 hours of growth (these cells were allowed to reach stationary phase at night time). (A) Visualisation and selection of cells in function of light scatter angles. The flow cytometer counted and displayed 500,000 particles. A heat map indicated the population density of these particles. Cells were selected (here 466,255 cells were encircled in region of interest 1). (B) Visualisation and selection of cells in function of their green fluorescence. PAL + yfp − cells were encircled in region of interest 2 whereas PAL − yfp + cells were encircled in region of interest 7. (C) Number of cells in function of fluorescence when gated by region of interest 1. The cells selected in region of interest 1 in panel A were separated here in function of their fluorescence so that it was possible to evaluate the number of PAL + yfp − cells indicated in region of interest 5 and PAL − yfp + cells indicated in region of interest 6. (D) Number of PAL + yfp − cells in the population. The cells selected in region of interest 2 in panel B were separated in function of their fluorescence so that it was possible to calculate the number of PAL + yfp − cells indicated in region of interest 4. (E) Number of PAL − yfp + cells in the population. The cells selected in region of interest 7 in panel B were separated in function of their fluorescence so that it was possible to calculate the number of PAL − yfp + cells indicated in region of interest 3. Characteristics of regions of interest are indicated under each panel; the numbers of cells per microliter of culture (Evt/μl) were used for subsequent data analyses. (TIF) [file pone.0110784.s001.tif]

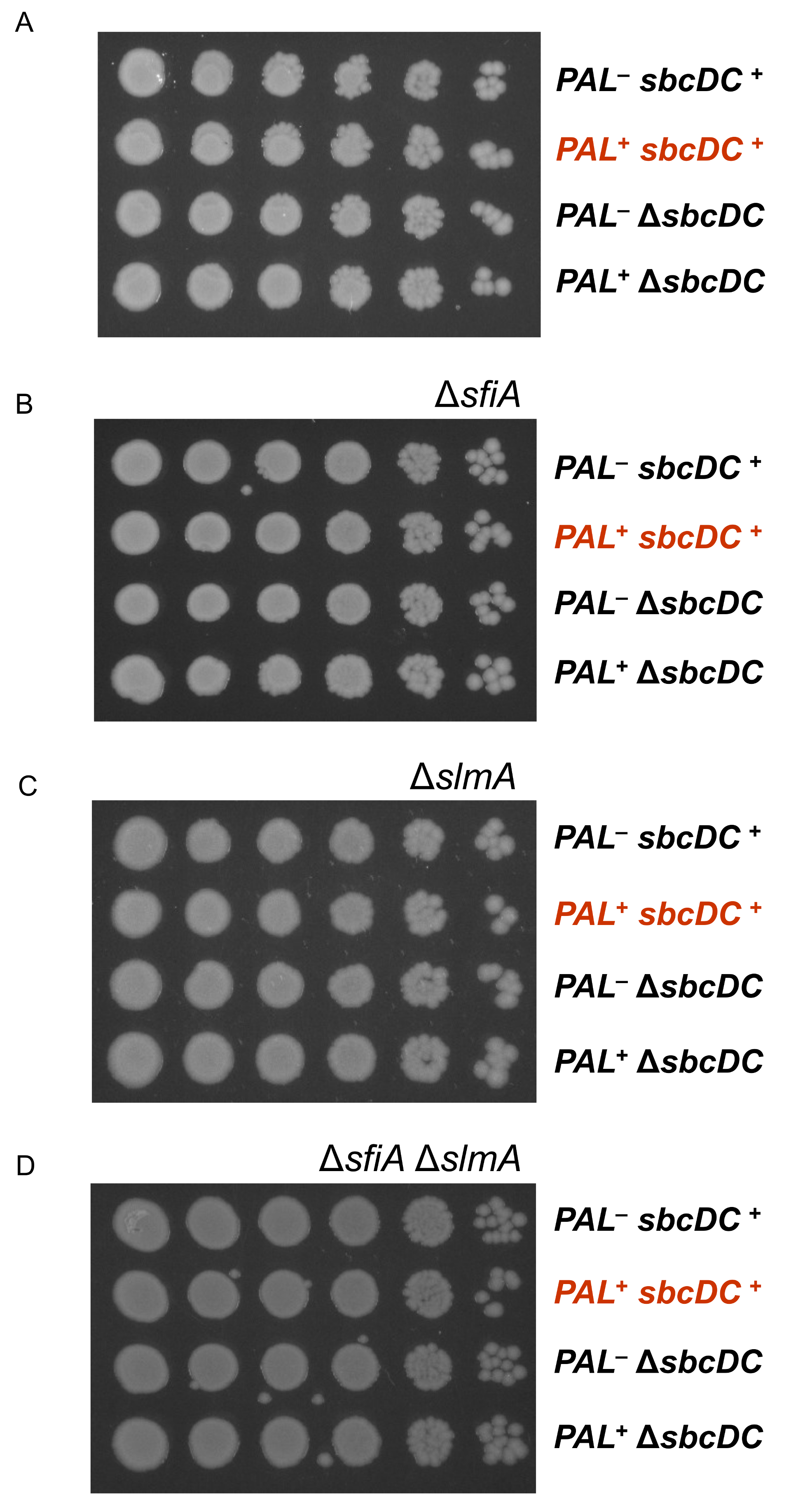

Supplement: Figure S2 — E. coli viability is not significantly affected by a chronic DSB. Viability of sbcDC + and ΔsbcDC E. coli strains containing or not the chromosomal 246 bp interrupted palindrome (PAL). Spot tests of ten-fold dilution series were carried out on LB plates. (A) Wild-type background strain. (B) ΔsfiA background strain. (C) ΔslmA background strain. (D) ΔsfiA ΔslmA background strain. (TIF) [file pone.0110784.s002.tif]
