# Supplementary material for: Repair on the Go: E. coli Maintains a High Proliferation Rate while Repairing a Chronic DNA Double-Strand Break
Source: PLoS One. 2014 Oct 29;9(10):e110784. doi: 10.1371/journal.pone.0110784 (PMC4213011; doi:10.1371/journal.pone.0110784)
Supplement: File S1 — Main supporting information file. This file includes additional materials and methods (strains and plasmids, spot test, time-lapse microscopy), additional results (time-lapse microscopy), Table S1 (E. coli strains), Table S2 (plasmids), Table S3 (oligonucleotides) and additional references. (DOC) [file pone.0110784.s003.doc]

## Additional Materials and Methods

**Strains and plasmids**

Lists of strains, plasmids and oligonucleotides used in this study can be found in Tables S1, S2 and S3, respectively. The SOS reporter plasmid pGB150 was constructed by ligating a 133 bp fragment representing the *sfiA* promoter (1,020,276-1,020,143 bp on the MG1655 chromosome) into the multiple cloning site of pGFPmut3.1, followed by sub-cloning of the XbaI-EagI fragment into pACYC184. The resulting chloramphenicol resistant plasmid contains a P*_sfiA_-gfp* fusion.

The pMH9 plasmid, also called pHM, is a pTOF24 derivative from which the PstI-SalI fragment of 1293 bp was excised and replaced by a 63 bp-polylinker containing PstI, BamHI, PacI, SacI, SmaI, SwaI, NheI, NotI, AscI and SalI restriction sites. The plasmid pDL1573 (pMH9-*sfiA*) was created in order to delete the *sfiA* gene by plasmid mediated gene replacement . Primer pairs used for the cross-over PCR on MG1655 genomic DNA were SfiA1-fwd/SfiA1-rev and SfiA2-fwd/SfiA2-rev. These primers permit the insertion of an XbaI site between the two homology arms. The cross-over PCR fragment was cloned in pMH9 using PstI and NheI restriction enzymes. The plasmid pDL5481 (pTOF-Δ*slmA*) was created in order to delete the *slmA* gene by plasmid mediated gene replacement. Primer pairs used for the cross-over PCR on MG1655 genomic DNA were slmAF1/slmAR1 and slmAF2/slmAR2. This fragment was cloned in pTOF24 using PstI and SalI restriction enzymes.

**Spot test**

After growth overnight of each strain at 37ºC under agitation in liquid LB medium, 10-fold serial dilutions were prepared and 3 μl of these dilutions were spotted onto LB agar plates. This experiment was carried out at least three independent times, giving similar results.

**Time-lapse microscopy**

After an overnight culture, *sbcDC***^+^** or Δ*sbcDC* cells containing or not the chromosomal 246 bp interrupted palindrome were diluted and these cultures were grown at 37°C in LB medium until an OD_600nm_ between 0.1 and 0.3. Then, 5 μl of cells were placed onto an airtight LB-coated slide. Brightfield images were acquired every 30 seconds for 2 hours at a resolution of 0.129μm per pixel using a Zeiss Axiovert 200 fluorescence microscope equipped with a Photometrics cool-SNAP HQ CCD camera. Images were analyzed and films were constructed using the program MetaMorph 6-3r2 (Molecular Devices).

**Results**

**Videos: Time-lapse microscopy shows that a chronic DSB delays cell division**

Time-lapse microscopy confirms the observations described in the main text. The *sbcDC***^+^** cells containing the 246 bp palindrome showed delayed division compared to the cells that are not subjected to a DSB per replication cycle (Video S1 compared to S2, S3 and S4). Importantly, the filamentation phenotype was dramatically exacerbated by the condition of the time-lapse experiment.

**Tables**

**Table S1: *E. coli* strains**

| **Strain** | **Genotype** | **Origin or construction** | **Figure(s), Table(s) and video(s)** |
| --- | --- | --- | --- |
| BT346 | *lexA3*ind *recAo^C281^* *srl350*::Tc^R^ |  |  |
| DL1777 | MG1655 *lacZ*χ- *lacI*^q^ |  | Figures 2A, 3, 4, 5, 6A, S2A, Tables 1, 2, 3, 4, 5, video S2 |
| DL2006 | BW27784 ΔP*_sbcDC_* P*_BAD_-sbcDC lacZ*::*PAL246 cynX*::Gm^R^ |  | Figure 1 |
| DL2151 | MG1655 *lacZ*χ- *lacI*^q^ Δ*sbcDC* |  | Figures 2A, 3, 5, 6A, S2A, Tables 1, 2, 3, 4, 5, video S4 |
| DL2573 | BW27784 ΔP*_sbcDC_* P*_BAD_-sbcDC lacZ^+^ cynX*::Gm^R^ |  | Figure 1 |
| DL2859 | MG1655 *lacZ*χ- *lacI*^q^ *lacZ*::*PAL246* *cynX*::Gm^R^ |  | Figures 2A, 3, 4, 5, 6A, S1, S2A, Tables 1, 2, 3, 4, 5, video S1 |
| DL2874 | MG1655 *lacZ*χ- *lacI*^q^ *lacZ*::*PAL246* *cynX*::Gm^r^ Δ*sbcDC* |  | Figures 2A, 3, 5, 6A, S2A, Tables 1, 2, 3, 4, 5, video S3 |
| DL3602 | BW27784 ΔP*_sbcDC_* P*_BAD_-sbcDC lacZ^+^ cynX*::Gm^R^ *lexA3*ind *malE*::Tn*5*(Km^R^) | DL2573 x P1 N1630 *lexA3*ind *malE::*Tn*5* | Figure 1 |
| DL3603 | BW27784 ΔP*_sbcDC_* P*_BAD_-sbcDC lacZ*::*PAL246 cynX*::Gm^R^ *lexA3*ind *malE*::Tn*5*(Km^R^) | DL2006 x P1 N1630 *lexA3*ind *malE::*Tn*5* | Figure 1 |
| DL3606 | BW27784 ΔP*_sbcDC_* P*_BAD_-sbcDC lacZ^+^ cynX*::Gm^R^ *lexA3*ind *malE*::Tn*5*(Km^R^) *lexA3* *recAo^C281^* *srl350*::Tc^R^ | DL3602 x P1 BT346 *recAo^C281^* *spl350*::Tc^R^ | Figure 1 |
| DL3607 | BW27784 ΔP*_sbcDC_* P*_BAD_-sbcDC lacZ*::*PAL246 cynX*::Gm^R^ *lexA3*ind *malE*::Tn*5*(Km^R^) *lexA3* *recAo^C281^* *srl350*::Tc^R^ | DL3603 x P1 BT346 *recAo^C281^* *spl350*::Tc^R^ | Figure 1 |
| DL4127 | MG1655 *lacZ*χ- *lacI*^q^ Δ*sfiA* | DL1777 PMGR using pDL1573 (pMH9sfiA) | Figures 6B, S2B, Tables 1, 2, 3, 4, 5 |
| DL4128 | MG1655 *lacZ*χ- *lacI*^q^ Δ*sbcDC* Δ*sfiA* | DL2151 PMGR using pDL1573 (pMH9sfiA) | Figures 6B, S2B, Tables 1, 2, 3, 4, 5 |
| DL4129 | MG1655 *lacZ*χ- *lacI*^q^ *lacZ*::*PAL246* *cynX*::Gm^R^ Δ*sfiA* | DL2859 PMGR using pDL1573 (pMH9sfiA) | Figures 6B, S2B, Tables 1, 2, 3, 4, 5 |
| DL4130 | MG1655 *lacZ*χ- *lacI*^q^ *lacZ*::*PAL246* *cynX*::Gm^R^ Δ*sbcDC* Δ*sfiA* | DL2874 PMGR using pDL1573 (pMH9sfiA) | Figures 6B, S2B, Tables 1, 2, 3, 4, 5 |
| DL5402 | MG1655 *lacZ*χ- *lacI*^q^ *intC*::λP_R_-*eyfp* (Cm^R^) | DL1777 x P1 RJA002 *intC*::λP_R_-*eyfp* (Cm^R^) | Figures 4, S1 |
| DL5403 | MG1655 *lacZ*χ- *lacI*^q^ *lacZ*::*PAL246* *cynX*::Gm^R^ *intC*::λP_R_-*eyfp* (Cm^R^) | DL2859 x P1 RJA002 *intC*::λP_R_-*eyfp* (Cm^R^) | Figure 4 |
| DL5606 | MG1655 *lacZ*χ- *lacI*^q^ Δ*slmA* | DL1777 PMGR using pDL5481 (pTOF-Δ*slmA*) | Figures 2B, 6C, S2C, Tables 1, 2, 3, 4, 5 |
| DL5607 | MG1655 *lacZ*χ- *lacI*^q^ Δ*sbcDC* Δ*slmA* | DL2151 PMGR using pDL5481 (pTOF-Δ*slmA*) | Figures 2B, 6C, S2C, Tables 1, 2, 3, 4, 5 |
| DL5608 | MG1655 *lacZ*χ- *lacI*^q^ *lacZ*::*PAL246* *cynX*::Gm^R^ Δ*slmA* | DL2859 PMGR using pDL5481 (pTOF-Δ*slmA*) | Figures 2B, 6C, S2C, Tables 1, 2, 3, 4, 5 |
| DL5609 | MG1655 *lacZ*χ- *lacI*^q^ *lacZ*::*PAL246* *cynX*::Gm^R^ Δ*sbcDC* Δ*slmA* | DL2874 PMGR using pDL5481 (pTOF-Δ*slmA*) | Figures 2B, 6C, S2C, Tables 1, 2, 3, 4, 5 |
| DL5622 | MG1655 *lacZ*χ- *lacI*^q^ Δ*sfiA* Δ*slmA* | DL4127 PMGR using pDL5481 (pTOF-Δ*slmA*) | Figures 6D, S2D, Tables 1, 2, 3, 4, 5 |
| DL5623 | MG1655 *lacZ*χ- *lacI*^q^ Δ*sbcDC* Δ*sfiA* Δ*slmA* | DL4128 PMGR using pDL5481 (pTOF-Δ*slmA*) | Figures 6D, S2D, Tables 1, 2, 3, 4, 5 |
| DL5624 | MG1655 *lacZ*χ- *lacI*^q^ *lacZ*::*PAL246* *cynX*::Gm^R^ Δ*sfiA* Δ*slmA* | DL4129 PMGR using pDL5481 (pTOF-Δ*slmA*) | Figures 6D, S2D, Tables 1, 2, 3, 4, 5 |
| DL5625 | MG1655 *lacZ*χ- *lacI*^q^ *lacZ*::*PAL246* *cynX*::Gm^R^ Δ*sbcDC* Δ*sfiA* Δ*slmA* | DL4130 PMGR using pDL5481 (pTOF-Δ*slmA*) | Figures 6D, S2D, Tables 1, 2, 3, 4, 5 |
| MRR | MC4100 *galK*::λP_R_-*cfp* (Amp^R^) *intC*::λP_R_-*eyfp* (Cm^R^) |  |  |
| MG1655 | F^-^ 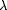^-^ *ilvG^-^ rfb-50 rph-1* |  |  |
| N1630 | *lexA3*ind *malE*::Tn*5*(Km^R^) |  |  |
| RJA002 | MG1655 *intC*::λP_R_-*eyfp* (Cm^R^) | MG1655 x P1 MRR *intC*::λP_R_-*eyfp* (Cm^R^) |  |

Abbreviations: Amp^R^ (ampicillin resistant at 100 μg/ml); Km^R^ (kanamycin resistant at 50 μg/ml); Gm^R^ (gentomycin resistant at 10 μg/ml); Tc^R^ (tetracycline resistant at 20 μg/ml); *lacZ*χ- (a χ site was removed from the *lacZ* gene); *PAL246* (a palindrome of 246 bp with a 24 bp interruption). NB. Please note that this MG1655 and all its derivatives also have an *fnr-267* mutation .

Table S2: Plasmids

| **Plasmid** | **Brief description** | **Source or Reference** |
| --- | --- | --- |
| pDL1573  (pMH9sfiA) | pMH9 derivative containing Δ*sfiA* knock-out fragment, Cm^R^ Ts Suc^S^ | This work |
| pDL5481  (pTOF-Δ*slmA*) | pTOF24 derivative containing Δ*slmA* knock-out fragment, Cm^R^ Ts Suc^S^ | This work |
| pACYC184 | Cm^R^ Tc^R^ |  |
| pGB150 | pACYC184 derivative containing the *gfp* gene under the control of the *sfiA* promoter; Cm^R^ | This work |
| pGFPmut3.1 | Amp^R^ | Clontech |
| pMH9  (pHM) | pTOF24 derivative containing a polylinker PstI-BamHI-PacI-SacI-SmaI-SwaI-NheI-NotI-AscI-SalI, Cm^R^ Ts Suc^S^ | This work |
| pTOF24 | Cm^R^ Km^R^ Ts Suc^S^ |  |

NB. Abbreviations: Amp^R^; ampicillin resistant (100 μg/ml), Cm^R^; chloramphenicol resistant (50 μg/ml), Km^R^; kanamycin resistant (50 μg/ml), Tc^R^; tetracycline resistant (15 μg/ml).Ts; temperature sensitive origin of replication (growth at 30°C), Suc^S^; sucrose sensitive (5% w/v).

Table S3: Oligonucleotides

| **Oligonucleotide** | **5’ – 3’ sequence** |
| --- | --- |
| SfiA1-fwd | AAAAACTGCAGCGCCATAGACTTTCATCAACC |
| SfiA1-rev | TAAATTTACTTAATGATACAAATTAGAGTGAATTCTAGATGAAGTGTACATAATCAATCCAGC |
| SfiA2-fwd | CTCACAGGGGCTGGATTGATTATGTACACTTCATCTAGAATTCACTCTAATTTGTATCATTAA |
| SfiA2-rev | TTTTTGCTAGCCAGCCCAGTTTAGCACCAGT |
| slmAF1 | AAAAACTGCAGCGATTCATATTGCCGATCCT |
| slmAR1 | CATCCGGCGTCATATTACTGCATGTTACAAAATACCCCTGAAAA |
| slmAF2 | CAGGGGTATTTTGTAACATGCAGTAATATGACGCCGGATG |
| slmAR2 | AAAAAGTCGACCCTGCCGTACTGCTTTAACC |

NB. Underlined sequences represent restriction recognition sequences.

## References

1. Merlin C, McAteer S, Masters M (2002) Tools for characterization of *Escherichia coli* genes of unknown function. J Bacteriol 184: 4573-4581.

2. Thoms B, Wackernagel W (1998) Interaction of RecBCD enzyme with DNA at double-strand breaks produced in UV-irradiated *Escherichia coli*: requirement for DNA end processing. J Bacteriol 180: 5639-5645.

3. Eykelenboom JK, Blackwood JK, Okely E, Leach DR (2008) SbcCD causes a double-strand break at a DNA palindrome in the *Escherichia coli* chromosome. Mol Cell 29: 644-651.

4. Elowitz MB, Levine AJ, Siggia ED, Swain PS (2002) Stochastic gene expression in a single cell. Science 297: 1183-1186.

5. Blattner FR, Plunkett G, 3rd, Bloch CA, Perna NT, Burland V, et al. (1997) The complete genome sequence of *Escherichia coli* K-12. Science 277: 1453-1462.

6. Lloyd RG, Buckman C, Benson FE (1987) Genetic analysis of conjugational recombination in *Escherichia coli* K12 strains deficient in RecBCD enzyme. J Gen Microbiol 133: 2531-2538.

7. Soupene E, van Heeswijk WC, Plumbridge J, Stewart V, Bertenthal D, et al. (2003) Physiological studies of *Escherichia coli* strain MG1655: growth defects and apparent cross-regulation of gene expression. J Bacteriol 185: 5611-5626.

8. Chang AC, Cohen SN (1978) Construction and characterization of amplifiable multicopy DNA cloning vehicles derived from the P15A cryptic miniplasmid. J Bacteriol 134: 1141-1156.
